# Inflammatory biomarkers in serum in subjects with and without work related neck/shoulder complaints

**DOI:** 10.1186/1471-2474-15-103

**Published:** 2014-03-26

**Authors:** Anna Matute Wilander, Monica Kåredal, Anna Axmon, Catarina Nordander

**Affiliations:** 1 Division of Occupational and Environmental Medicine, Skåne University Hospital, Lund SE-221 85, Sweden

**Keywords:** Work related, Musculoskeletal disorders, Inflammation, Biomarker, Cytokine

## Abstract

**Background:**

Although it has recently been recognised that inflammation is important in the development of work-related musculoskeletal disorders (MSDs), the exact pathophysiological pathways are unknown.

**Methods:**

We investigated serum concentrations of inflammatory cytokines in 35 female supermarket cashiers with repetitive work tasks and work related neck/shoulder complaints, compared with those from 25 women without MSDs (6 supermarket cashiers and 19 middle-school teachers or faculty staff). None of the subjects were pregnant or lactating, and showed no signs of rheumatoid arthritis, systemic lupus erythematosus, cancer, diabetes, coronary artery disease or inadequately controlled hypertension. Serum levels of IL-1α, IL-1β, IL-6, IL-8, IL-10, IL-12, MCP-1, MIP-1α, MIP-1β, TNF-α, GM-CSF, CTGF and CRP were analysed.

**Results:**

The women with pain related to MSD had higher serum concentrations of MIP-1β (median, 25th-75th percentile: 90.0 pg/mL, 62.5-110 vs. 73.1 pg/mL, 54.6-88.3; p = 0.018), IL-12 (0.26 pg/mL, 0.26-0.26 vs. 0.26 pg/mL, 0.26-0.26; p = 0.047) and CRP (0.5 mg/L, 0.5-1.6 vs. 0.5 mg/L, 0.5-0.5; p = 0.003), than control subjects. Levels of MIP-1α, MIP-1β and CRP were correlated with the reported intensity of neck/shoulder pain (r = 0.29, p = 0.03 for MIP-1α; r = 0.29, p = 0.02 for MIP-1β and r = 0.43, p = 0.001 for CRP). No statistically significant differences in serum levels were found for the remaining cytokines.

**Conclusions:**

Otherwise healthy females with ongoing work-related neck/shoulder pain showed higher serum concentrations of MIP-1β, IL-12 and CRP than controls, and the levels of MIP-1α, MIP-1β and CRP were correlated to pain intensity. These results support previous findings that inflammatory processes play a part in work related MSDs.

## Background

Work-related musculoskeletal disorders (MSDs) are a widespread public health problem. However, the exact pathophysiological pathways of these disorders are still unknown. It is therefore important to study possible mechanisms, to enable the early identification of those at risk of developing MSDs as a result of different types of exposures, and to allow appropriate therapeutic interventions.

The role of inflammation in the early phases of MSDs has been highlighted in many recent publications [1,2]. Several animal studies have shown inflammatory changes in tissues following repetitive tasks. Soslowsky et al. [3] reported continuous deterioration of the mechanical properties of a tendon, induced by overuse for 16 weeks. Histological examination revealed increased cellularity and increased cross-sectional area of the tendon, indicating inflammation. Signs of widespread inflammation have also been found in the tissues and in serum of animals performing repetitive tasks. Barbe et al. [4] constructed an animal model in which rats were trained to reach repeatedly for food pellets, or to reach and pull a handle for a food reward [2,5]. Although this was a low-intensity task, increasing numbers of infiltrating macrophages were seen in all four limbs after one month of performing the task, indicating inflammation on a systemic level. A significant increase in resident macrophages in the limb used for reaching, and in serum levels of the pro-inflammatory cytokine IL-1α were found after eight weeks. Barbe et al. therefore suggested that musculotendinous injuries resulting from performing repetitive, and/or forceful, tasks lead to an early inflammatory response. In a more recent study of humans, Carp et al. [6] found a correlation between the severity of newly developed upper extremity overuse MSD in patients and serum levels of inflammatory cytokines and C-reactive protein (CRP), thus also supporting the role of an early inflammatory response in the development of overuse MSD. If the loads are low, or sufficient time is allowed, complete recovery from inflammation may be possible. However, if tissue repair cannot take place due to continued demands on injured and inflamed tissues, a chronic inflammatory process may result [1,7,8].

If systemic inflammatory processes do indeed play an important role in the development of overuse injuries, it may be possible to assess work-related MSDs using biomarkers of inflammation. Such biomarkers provide objective measures of pathophysiological processes, and studies in humans at different stages of disease progression could provide valuable information. The aim of the present study was, thus, to investigate whether levels of biomarkers of inflammation were higher in workers with work-related MSDs, recruited from the working population, and not from a health-care provider, than in females not reporting any MSDs. In addition, since previous studies have shown an increased number of macrophages in tissues of animals performing repetitive tasks [4,9], cytokines known to be associated with macrophages were also analysed.

## Methods

### Study design

Subjects were recruited from two ongoing epidemiological studies (Balogh et al. to be published, Arvidsson et al. to be published), in which postal questionnaires regarding the frequency and intensity of musculoskeletal symptoms [10], as well as whether the symptoms were perceived as work-related, were sent out. Female supermarket cashiers with and without ongoing work-related neck and/or shoulder pain were identified, as were female teachers without such complaints. Cashiers are of special interest since they are exposed to repetitive reaching and grasping during a substantial part of the working day. Teaching, on the other hand, is essentially a varied, mobile form of work. To ensure a sufficiently large control group, faculty and staff from our department, all performing varied work tasks, were also included. Eligible subjects received a letter containing information about the study and were thereafter contacted by telephone. Those who were willing to participate in the study were interviewed regarding their medical health status, using a standardized questionnaire. A final pain assessment, and blood sampling, were performed at our clinic.

The study was approved by the Regional Ethical Review Board in Lund. All subjects signed an informed consent form, after receiving written and oral information.

### Pain assessment

For MSD subjects inclusion criteria were pain or discomfort in the neck and/or shoulders since at least one month, as reported in the questionnaire. The intensity of pain was required to be at least 3 on a Borg scale between 0 and 10 [11,12]. During the past 12 months, the subject was required to have experienced this pain “often” or “very often” on a 5-point scale ranging from “never”, “seldom”, “sometimes”, “often” to “very often” [13]. The problems were also to be perceived by the subject as work related. On the day of blood sampling, the intensity of pain was required to be at least 3.

Immediately following blood sampling, all participants were interviewed regarding musculoskeletal symptoms in the neck, shoulders, elbows and hands, upper and lower back, hips, knees and feet, providing data on pain or discomfort in 9 different regions of the body. The MSD subjects were once again asked to estimate the intensity of pain in their neck and shoulders, using the 11-point Borg scale. An average neck and shoulder pain intensity was calculated from the reported intensities in these regions. A standardized physical examination of the neck, shoulders, elbows and hands was also performed in and diagnoses were made according to predefined criteria [14,15].

Blood samples were collected from the individuals in the control group, provided that they had reported pain or discomfort from any site in the musculoskeletal system no more than “sometimes” during the past 12-months, and did not report any musculoskeletal pain at all on the day of the blood sampling.

### Subjects

Exclusion criteria were known rheumatoid arthritis or systemic lupus erythematosus. Subjects with cancer, diabetes, coronary artery disease or inadequately controlled hypertension were also excluded, as were women who were pregnant or lactating. The body mass index (BMI) was required to be between 18 and 30. Subjects were excluded if they were currently taking medication containing cortisone. Subjects taking non-steroidal anti-inflammatory drugs (NSAIDs) were included only if they could refrain from taking these medications for 7 days prior to the blood test. Similarly subjects using tobacco products were excluded unless they could refrain from using such products 7 days prior to the blood test. Subjects who had had a cold or any other infection during the 7 days prior to the blood test were asked to come back at a later date. On the day of the examination and blood sampling, blood samples were also taken to determine thyroid function and rheumatoid factor (RF). Subjects showing abnormalities regarding these parameters were excluded.

Fifty-one subjects with work-related neck and/or shoulder pain were recruited for blood sampling. Of these, one subject who tested positive for RF and one subject found to have thyroid dysregulation were excluded. Fourteen subjects did not report neck and/or shoulder pain of at least moderate intensity, and were therefore excluded, leaving a group of 35 subjects.

Thirty-six symptom-free control subjects were recruited. Of these, 11 reported temporary pain on the day of blood sampling and were excluded, leaving 25 control subjects in the group. Of the control subjects 6 were cashiers and 19 were middle-school teachers or faculty and staff at our department, with non-repetitive work tasks.

The mean BMI was 24 kg/m^2^ (SD 3) in the MSD subjects, and in the control group 23 kg/m^2^ (SD 2). BMIs were evenly distributed between subjects with different work tasks in the control group. The mean age was 45 years (SD 14) in the MSD group and 42 (SD 10) in the control group, also evenly distributed between subjects with different work tasks.

### Serum samples and analyses

All serum samples were collected prior to the physical examination, between 10 am and 12 noon. Samples were either sent to the laboratory at the Department of Clinical Chemistry at the Skåne University Hospital for immediate analysis or stored at -80°C until analysis. Serum samples for the analysis of thyroid function (thyroid stimulating hormone and thyroxine) as well as RF and C-reactive protein (CRP) were sent continuously to the laboratory at the Department of Clinical Chemistry, and analysed according to standard procedures.

Cytokine serum levels of 6 interleukins [IL-1α, IL-1β, IL-6, IL-8, IL-10, IL-12 (p70)], monocyte chemotactic protein 1 (MCP-1), macrophage inflammatory protein 1α (MIP-1α), MIP-1β, granulocyte-macrophage colony-stimulating factor (GM-CSF) and tumour necrosis factor α (TNF-α) were determined using multiplex immunoassay analysis, on a Bio-Plex 200 Luminex instrument (Bio-Rad, Hercules, CA, USA). Triplicate analyses were performed on each sample. Single-plex magnetic bead-based assays (Bio-Rad) containing beads coated with antibodies specific for each analyte were mixed, and the assay was performed according to the instructions of the manufacturer’s instructions, with some minor changes. The time for incubation between beads and sample was increased to 60 minutes. All wash steps were performed using a Bio-Plex Pro wash-station (Bio-Rad). Standard curve and concentrations of cytokines were determined by Bio-Plex Manager 6.0 software (Bio-Rad) using five-parameter curve fitting. The lower limit of detection (LOD) was determined as the mean value of the concentration of the lowest detectable standard identified within each analysis. The LODs for the measured cytokines were (pg/mL): IL-1α, 0.30; IL-1β, 0.52; IL-6, 0.30; IL-8, 0.41; IL-10, 0.43; IL-12, 0.51; TNF-α, 1.2; MIP-1α, 0.32; MIP-1β, 0.86; MCP-1, 0.36; GM-CSF, 0.85.

The levels of a few some of the cytokines were very low, partly due to dilution of the samples (performed according to the instructions of the protocol), and values were extrapolated (flagged by the software) below the lowest standard point. After multiplying by four, to calculate the true concentration, most values were above the detection limit. Concentrations that were still below the LOD were assigned a value of half the LOD. The lower detection level of CRP was 1.0 mg/L, and values below this were set to 0.5 mg/L.

Connective tissue growth factor (CTGF) was analysed using a commercially available ELISA kit from Adipo Bioscience (Santa Clara, CA, USA). Serum samples were analysed in duplicate according to the manufacturer’s instructions.

### Statistical analysis

All statistical analyses were conducted in SPSS 18.0 (SPSS, Chicago, IL, USA). A p-value of less than 0.05 (two-tailed) was accepted as a statistically significant difference. Since the data were skewed, comparisons between group means were performed using independent samples t-tests with values transformed according to the natural logarithm, giving normally distributed data. To investigate the effects of possible confounders ANOVA analysis was applied for the covariates BMI and age. Correlations were tested using Spearman’s test.

## Results

All the cytokines except IL-1α and GM-CSF could be detected in the serum of the majority of the subjects. When analysing the cytokines we found correlations between TNF-α and the interleukins IL-1β, IL-6 and IL-10 (Table 1). Correlations were also found between the interleukins themselves, although these were not as strong. MIP-1β was weakly correlated with TNF α, IL-1β and IL-6. MIP-1α showed a statistically significant correlation to IL-12.

**Table 1:** **Correlation matrix (Spearman rank-correlation coefficients; r**_**s**_**) for cytokine and CRP levels**

		**IL-6**	**IL-8**	**IL-10**	**IL-12**	**MCP-1**	**MIP-1α**	**MIP-1β**	**TNF-α**	**CTGF**	**CRP**^**1**^
IL-1β	r_s_	.34**	.04	.57**	.07	.01	.13	.31*	.70**	.05	.01
IL-6	r_s_		-.08	.34**	-.12	.10	-.07	.16	.62**	-.21	.08
IL-8	r_s_			.12	-.13	.36**	.12	.34**	.03	-.28*	.01
IL-10	r_s_				.01	.05	.02	.24	.61**	-.04	-.15
IL-12	r_s_					-.03	.44**	.09	-.03	.09	-.19
MCP-1	r_s_						-.11	.05	.10	-.17	.12
MIP-1α	r_s_							.16	.01	-.11	.12
MIP-1β	r_s_								.33**	-.15	.01
TNF-α	r_s_									-.12	-.18
CTGF	r_s_										-.14

Results in bold face are statistically significant.

^1^CRP value from one individual is missing due to technical errors.

The results obtained from the serum analyses are summarized in Table 2. A statistically significant increase in serum concentration of IL-12, MIP-1β and CRP was found in the MSD group compared to the control group. Furthermore, the concentrations of all the cytokines except MCP-1, TNF-α and CTGF were higher in the MSD group than in the control group, although these differences were not statistically significant.

**Table 2:** **Cytokine¹ (in pg/mL) and CRP (in mg/L) levels in subjects with work-related musculoskeletal disorder (MSD) and controls, given as median, 25**^**th **^**and 75**^**th **^**percentile**

	**Subjects with MSD (N=35)**	**Controls (N=25)**	**Crude p-value**	**Adjusted p-value**^**3**^
IL-1β	0.58 (0.26-0.90)	0.53 (0.26-0.62)	0.22	0.21
IL-6	0.82 (0.15-1.8)	0.50 (0.15-1.1)	0.14	0.19
IL-8	5.7 (3.8-8.1)	5.3 (3.9-7.0)	0.85	0.85
IL-10	1.0 (0.84-2.4)	0.93 (0.76-1.6)	0.17	0.13
IL-12	0.26 (0.26-0.26)	0.26 (0.26-0.26)	**0.047**	0.05
MCP-1	27 (20-40)	29 (22-38)	0.38	0.38
MIP-1α	0.16 (0.16-2.2)	0.16 (0.16-0.1)6	0.07	0.08
MIP-1β	90 (63-110)	73 (55-88)	**0.02**	**0.02**
TNF-α	3.1 (1.8-5.1)	3.2 (2.4-3.8)	0.81	0.65
CTGF	244 (67-420)	67 (67-350)	0.63	0.78
CRP^4^	0.50 (0.50-1.6)	0.50 (0.50-0.50)	**0.003**	**0.02**

P-values from T-tests^2^.

Results in bold face are statistically significant.

^1^IL-1α and GM-CSF were not detectable in the serum of any subject and are therefore not shown in the table.

^2^p-values calculated using values transformed according to the natural logarithm.

^3^Adjusted for age and BMI.

^4^CRP value from one individual is missing due to technical errors.

No evidence was found of a difference in inflammatory mediators when the control subjects were divided into two groups according to their working conditions, forming one group withvaried and non-repetitive work tasks (teachers and faculty and staff at our department), and another with repetitive work tasks (cashiers). When comparing the affected cashiers with the control subjects with non-repetitive work tasks, the former still showed higher levels of IL-12 (p = 0.047), MIP-1β (p = 0.04) and CRP (p = 0.002). The number of unaffected cashiers was too small to allow statistical testing.

**Figure 1:**
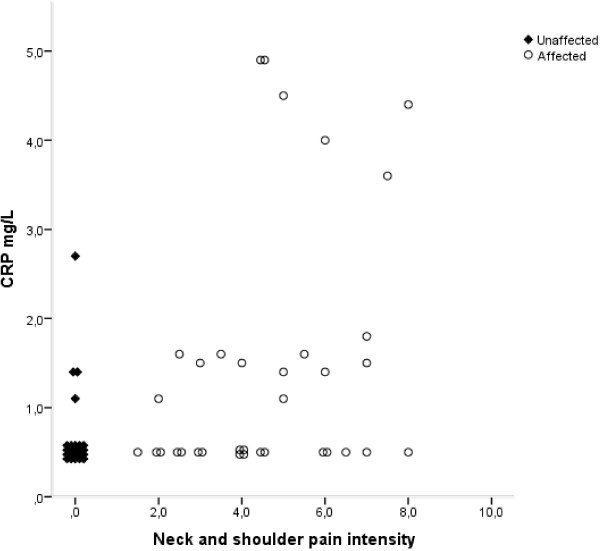
**Correlation between CRP and average neck/shoulder pain intensity.** Spearman’s rank correlation coefficient (r_s_) = 0.43; p = 0.001. Markers of subjects with the same value on both axes have been scattered around the true value, for the sake of clarity. The CRP concentration is missing for one individual due to a technical error. Open circles denote subjects with work-related MSD (affected), and filled diamonds denote control subjects (unaffected).

In the MSD group the median of the self-reported pain intensity from the neck and shoulder region was 4.5 (25^th^-75^th^ percentile 3.0-6.0) and the median duration of pain from this region was 72 months (36-120). The median number of diagnoses was 1.0 (0-2.5) in the neck and shoulder region and 1.0 (0-3.0) in the neck/shoulder and elbow/hand region combined. The most frequent diagnoses were rotator cuff tendonitis, acromioclavicular joint syndrome and tension neck syndrome. A statistically significant positive correlation was found between pain intensity in the neck/shoulders and three cytokines: MIP-1α, MIP-1β and CRP (Figure 1). The correlation coefficients (r_s_) were 0.29 (p = 0.03), 0.29 (p = 0.02) and 0.43 (p = 0.001) for MIP-1 α, MIP-1β and CRP, respectively.

The median number of affected body regions in the MSD subjects, of the 9 regions from which data were collected, was 4.0 (2.0 – 5.0). CRP concentration was correlated with the total number of affected body regions (r_s_ = 0.45, p < 0.001).

No other cytokines showed correlations with pain intensity or the number of body regions affected. CRP concentration was positively correlated with BMI (r_s_ = 0.48, p < 0.001). However, we found no correlations between the cytokine levels and age or BMI. BMI was weakly correlated with pain intensity (p = 0.10) and the number of affected body regions (p = 0.06). Adjustment for BMI and age did not significantly affect the results (Table 2).

## Discussion

We found increased levels of IL-12, MIP-1β and CRP in the serum of subjects with work related MSD in the neck/shoulder region. We believe these findings represent an ongoing inflammation in these individuals.

### Methodological considerations

As there was a time delay between answering the initial postal questionnaire and blood sampling in some cases, all subjects were interviewed regarding their current health status on the day of the blood test, and only subjects who were affected or unaffected, at the time of answering the questionnaire and on the day of the blood test, were included in the study. All calculations were performed on the results obtained from the interview and examination on the day of the blood test. In this way we were able form clearly distinct groups of subjects with and without MSDs.

The serum used for analysing the cytokines was frozen immediately after sampling. Since the recruiting of suitable subjects was a lengthy process, some samples were stored in the freezer for up to three years. When analysing the samples, we therefore paid special attention to any signs of possible drift in concentrations in the older samples. However, we observed no such drift, and therefore believe that the storage time did not affect the quality of the samples or the results obtained. Blood tests for CRP, on the other hand, were analysed continuously at the laboratory of the Skåne University Hospital. This resulted in a relatively crude analysis for our purpose. In spite of this, we found a statistically significant elevation of CRP levels in the MSD group and a correlation between CRP and pain intensity.

The age distributions in the affected and unaffected groups were comparable, and we found no correlations between age and any of the cytokines analysed or CRP. Age has, however, been suggested to correlate with inflammatory status [16], and we therefore adjusted for this variable. Doing so did not significantly change the results.

Increased BMI is known to be associated with elevated CRP levels [17], and obesity has also been reported to affect cytokine levels [18] and, therefore, only subjects with BMI in the range 18-30 were included in the study. We found no correlations between the cytokines and BMI but, as would be expected, we did find a correlation between BMI and CRP. The two groups were, however, very similar with regard to BMI, and adjusting for BMI in a statistical model did not affect the result.

CRP is a marker of any type of inflammatory process, and there may be many reasons for an increase in CRP levels in an individual. Individuals with colds or other infections were, however, asked to return for blood sampling and examination at a later date. Our exclusion criteria also contained several disorders associated with inflammation. Thus, we believe that the elevated CRP levels found in the MSD group did in fact reflect an inflammatory component of their MSD.

IL-12 is released from classically activated macrophages following activation signals via e.g. TNF-α and GM-CSF [19]. IL-12 levels were generally low in this study, and only four subjects had levels above the LOD. However, all of these were MSD subjects and we therefore observed a statistically significant difference between groups with and without MSDs. This finding is interesting, but it is not possible to draw any conclusions with such a limited number of samples above the LOD.

Performing multiple analyses may increase the risk of discovering seemingly significant findings by chance. This is a limitation of the study, but the observed increases in CRP and MIP-1β are in line with previously published results, and can be explained from a pathophysiological perspective.

As expected, many of the cytokines analysed were found to be correlated with each other. This is consistent with the belief that inflammatory cytokines can stimulate the production of one another. For example, IL-1α and TNF-α have been shown to induce cytokine gene expression and production [20].

### Implications

We were not able to verify the results of previous studies, in which the pro-inflammatory cytokines IL-1 or TNF-α were found to be elevated in animals and patients suffering from MSD caused by repetitive stress [1,4,6]. In these studies, relatively newly developed disorders were examined, whereas our subjects had a long history of overuse MSD. However, increases were observed in pro-inflammatory chemokines (small cytokines), as discussed below.

Since previous studies have shown an increase in macrophages in the tissues, as well as an increase in the macrophage inflammatory protein 3a in serum, in animals performing repetitive tasks [5], we included cytokines associated with macrophages in our analyses. The results showed a statistically significant increase in MIP-1β, a cytokine released by neutrophils, which acts as a chemokine and activating factor for monocytes and macrophages [21]. This finding is consistent with the findings of Barbe et al. [4]. Interestingly, MIP-1β was also correlated with the reported intensity of pain in the neck and shoulder in our subjects, suggesting that the level of MIP-1β increases with the severity of the disorder. To the best of our knowledge, MIP-1β has not previously been evaluated in subjects with overuse MSD. MIP-1α was also correlated to pain intensity, and showed a tendency towards elevated serum levels in the MSD group. However, this elevation was not statistically significant.

CRP is a well-known and reliable biomarker of inflammation and tissue injury [17], and it has also been found to be elevated in subjects with repetitive work tasks [22] and MSD [6]. We found increased CRP levels in a substantial proportion (48%) of the affected subjects, indicating an underlying systemic inflammatory process in these individuals. Like MIP-1α and MIP-1β, CRP was correlated to the severity of neck/shoulder symptoms, but interestingly, MIP-1α, MIP-1β and CRP were not correlated with each other. It is possible that these biomarkers reflect different stages of an inflammatory process. The MSD subjects had both a long history of pain and a continuing exposure to repetitive work tasks. It could, therefore, be expected that we would find different stages of the inflammatory process within this group, and perhaps even within the same individual.

It has been suggested that repetitive and force demanding tasks lead to tissue fatigue failure [7,8], initially with inflammatory processes, followed by fibrotic processes. CTGF has been suggested as a potential mediator of fibrotic events [23,24], and has been shown to increase in the later stages of work-related MSDs [25,26]. Elevated levels of MIPs and CRP, but no difference concerning CTGF, between the subjects with and without MSD in the present study, may thus indicate that the affected subjects were in an inflammatory phase.

Since no differences were seen in cytokine levels between the control subjects with and without repetitive work tasks, we believe that the observed increase in inflammatory mediators seen in the MSD group is due to the musculoskeletal disorder resulting in pain, and not to the repetitive work itself.

## Conclusions

Signs of inflammation could be found in our subjects, despite the fact that they were recruited from the working population, and not from a health-care provider. We believe this is an important field for future research, since discovering the underlying pathophysiological mechanisms causing work-related MSD provide opportunities to assess, treat and prevent these disorders.

## Competing interests

The authors have no professional or financial affiliations that would bias this work.

## Authors’ contributions

CN participated in the study design and in the drafting of the manuscript. AMW collected and compiled the data and drafted the manuscript. MK was responsible for the chemical analyses; AA contributed to the statistical analyses. All authors read, contributed to and approved the final manuscript.

## Pre-publication history

The pre-publication history for this paper can be accessed here:

http://www.biomedcentral.com/1471-2474/15/103/prepub
